# A Novel Auto-Sorting System for Chinese Cabbage Seeds

**DOI:** 10.3390/s17040886

**Published:** 2017-04-18

**Authors:** Kuo-Yi Huang, Jian-Feng Cheng

**Affiliations:** Department of Bio-Industrial Mechatronics Engineering, National Chung Hsing University, Tai-Chung 402, Taiwan; b9844002@gmail.com

**Keywords:** Chinese cabbage seeds, machine vision, auto-sorting

## Abstract

This paper presents a novel machine vision-based auto-sorting system for Chinese cabbage seeds. The system comprises an inlet-outlet mechanism, machine vision hardware and software, and control system for sorting seed quality. The proposed method can estimate the shape, color, and textural features of seeds that are provided as input neurons of neural networks in order to classify seeds as “good” and “not good” (NG). The results show the accuracies of classification to be 91.53% and 88.95% for good and NG seeds, respectively. The experimental results indicate that Chinese cabbage seeds can be sorted efficiently using the developed system.

## 1. Introduction

In Taiwan, Chinese cabbage is a staple vegetable that can be cultivated all year round. Healthy seedlings from the seedling propagation station (nursery) are used to cultivate fields; therefore, seed quality is a crucial factor in growing seedlings. Image processing is a powerful and widely-used method for inspecting agricultural products. Rodriguez et al. [1] predicted the color, morphology, and appearance of grape seeds and grape berries using image processing to establish an objective browning index of seeds. Xu and Zhao [2] developed an automated strawberry grading system based on shape, size, and color features. Wiwart et al. [3] used image processing and principal component analysis to identify wheat varieties according to shape features (Feret's diameter, roundness, aspect ratio, solidity) and color descriptors of hue, saturation, and intensity (HSI) and L*a*b*. Tanabata et al. [4] designed high-throughput phenotyping software for measuring seed shape; a scanner was used to obtain rice and seed images. In this software, shape features such as circularity, length-to-width ratio, and length perimeters are calculated to detect rice varieties. The software can also classify seeds of other crops, but is limited by the image capture device, and does not allow for follow-up of the automated classification mechanism. ElMasry et al. [5] developed a fast and accurate computer-based machine vision system for detecting irregular potatoes in real time, using shape features and Fourier shape descriptors to detect regular and irregular potatoes. Their experimental results showed that the average accuracy of the system was 96.2%. Huang [6] developed a machine vision system for detecting diseases in Phalaenopsis seedlings, in which the features of the lesion area are extracted using Rayleigh transform and image processing, followed by the application of a detection line algorithm to estimate red, green, and blue (RGB) gray curves in the lesion area. A Bayes classifier is applied to detect and classify diseases.

Classifiers are also applied in agriculture. For example, Wang et al. [7] used hyperspectral images and neural networks to detect chilling injury in apples. An RGB image was converted into L*a*b* images, and five optimal wavelengths were determined. The average accuracy rate of the neural network model was 98.4%. Huang [8] presented an application for neural networks and image processing for evaluating and classifying the quality of areca nuts. Defects in areca nuts caused by diseases or insects were segmented through a detection line method. The principal axis length, secondary axis length, axis number, area, perimeter, compactness, and mean gray level of each nut image on the R, G, and B bands were used to detect the defects in areca nuts in the classification procedure. A back-propagation neural network classifier was employed to sort the quality of the nuts. Cheng et al. [9] used machine vision and neural networks to classify the quality of moldy peanuts. First, the Sobel filter was used to detect the image edge, and a complete image of the peanuts was obtained using dilation, erosion, and filling operators. Second, an HSI color model and gray co-occurrence matrix (entropy) served as the input neurons of the neural networks for quality classification. Huang et al. [10] used hyperspectral imaging and a least squares support vector machine (LSSVM) to classify maize seeds. Chaugule et al. [11] developed a new feature extraction method to extract the fusion of angle features for paddy-seed classification. Lurstwut and Pornpanomchai [12] presented a machine vision application for rice-seed germination analysis through image processing and on the basis of color, size, and texture features.

According to data from the Futian Nursery Grounds in Xizhou Township, Changhua County, Taiwan, the labor cost for determining the quality of Chinese cabbage seeds in Taiwan has recently increased by up to 50%. Consequently, farmers now face the problem of excessive costs, necessitating the development of a novel automatic sorting system for improving sorting processes for farmers. Therefore, the aim of this study was to design a machine vision system for sorting seeds. The technical goals were to develop an algorithm for extracting the shape, color, and texture features of seeds and to subsequently classify the seeds into different grades using the aforementioned features.

## 2. Materials and Methods 

### 2.1. Image Capture System and Experimental Samples 

**Figure 1:**
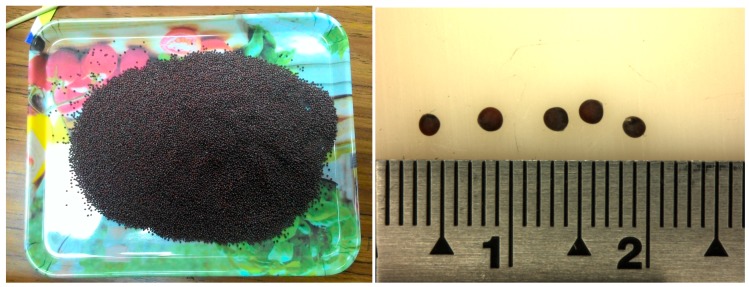
Chinese cabbage seeds.

The image capture system developed in this study includes a GigE CCD camera (DFK-23G274, Imaging Source Inc., Taipei, Taiwan), low-distortion lens (ML-MC25HR, SCHOTT MORITEX Inc., Saitama, Japan), diffuse ring light, and computer (Intel Core i7-2600 CPU, 2.78 GB RAM) to capture RGB color images measuring 1600×1200 pixels in bitmap format. The image processing software was developed in Visual Studio 2010-C# and EmguCVx86 2.4.0.1717. The Chinese cabbage seeds (CC-801; Figure 1) were provided by Futian Nursery Grounds.

### 2.2. Machine Vision System

**Figure 2:**
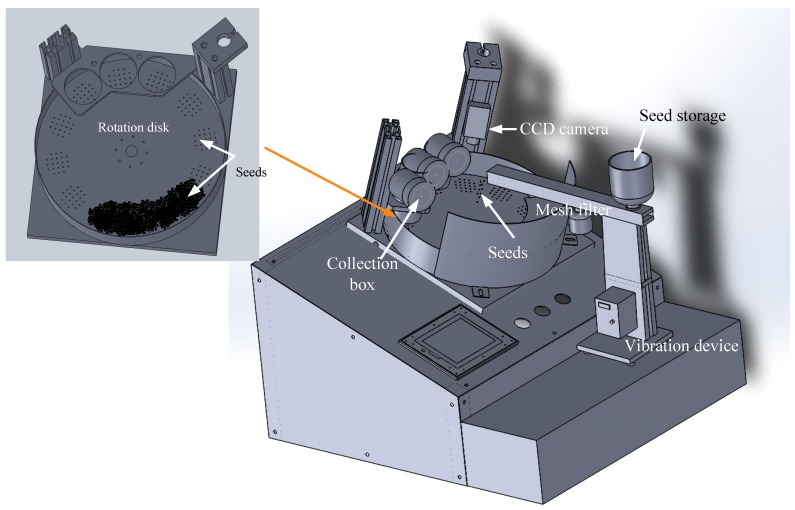
Configuration diagram of auto-sorting device for Chinese cabbage seeds.

The machine vision system implemented in this study includes a seed storage drum, mesh filter, rotation disk, outlet device, image capture system, collection box, programmable controller (PC), and programmable logic controller (PLC; Figure 2). First, the seeds were placed in the seed storage drum; they were placed on the rotation disk after small seeds and dust has been removed through the vibration device and mesh filter. The rotation disk has a total of 12 detection regions, each consisting of 21 holes. The field of view of the image is 40 mm × 40 mm. The seeds were sucked by the suction device under the rotation disk, captured on the detection region by using the CCD camera, and sorted using the proposed algorithms in real time. Second, the seeds were blown into the collection box in accordance with the sorting results.

### 2.3. Regional Segmentation and Feature Extraction

#### 2.3.1. Seed Image Segmentation

**Figure 3:**
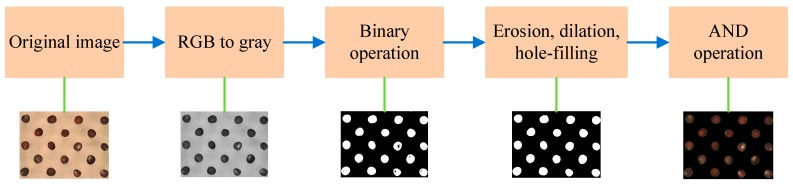
Image preprocessing.

Segmentation of the seed image after the features of the seeds have been extracted is an essential procedure. The steps include binary, erosion, dilation, and hole-filling operations [13] to remove noise and fill the holes in the seeds, thereby obtaining the entire binary image. Finally, the image of the entire seed can be obtained by applying the AND logic operator to the original and binary images. The segmentation steps and results in this study are shown in Figure 3. Each captured image contained 21 seeds. To extract the features of each seed, a sub-image of the seed must be segmented.

#### 2.3.2. Feature Extraction

Shape, texture, and color feature analysis have been employed extensively for classification. Shape is a vital indicator of seed quality. In a previous study, shape features [14,15,16] included compactness (*COM*), circularity (*CIR*), defect ratio (*DR*), elongation (*ELO*), ellipticity index (*EI*), eccentricity (*ECC*), convex hull, and symmetry area ratio (*SAR*). The mathematical formulations and definitions of the features are provided in Table 1.

A black or reddish-brown surface color indicates that a seed is in excellent condition, whereas a stale or low-quality seed appears gray or glaucous. In this study, RGB and HSI color models were used to detect seed quality. Color features such as the average gray level values of RGB and HSI bands were treated as input neurons in the neural networks. Textural features such as the angular second moment (ASM), entropy (ENT), contrast (CON), and homogeneity (HOM) from the gray level co-occurrence matrix (GLCM) [17] were employed to classify seeds as ”good” or “not good” (NG). Mathematical formulations of GLCM features are provided in Table 2. The GLCM is a square matrix (N×N), where *N* is the number of different gray levels in an image. An element P(i,j,d,θ) of the GLCM of an image represents the relative frequency, where *i* is the gray level at location (*x*, *y*) and *j* is the gray level of the neighboring pixel at a distance *d* and orientation *θ* from location (*x*, *y*). In this study, GLCMs with a distance of one pixel and orientations of 0°, 45°, 90° and 135° were used for sub-images.

**Figure 8:**
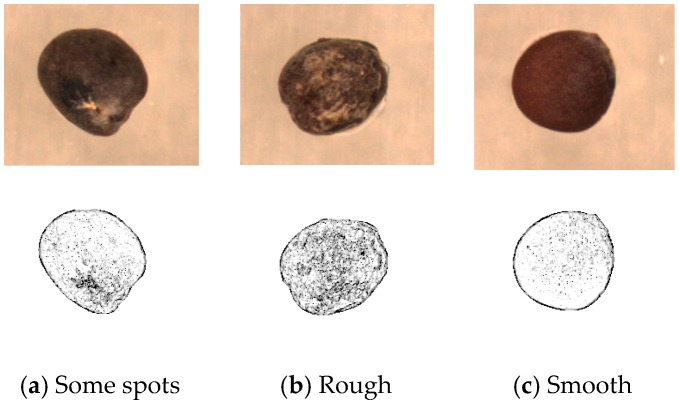
LSP coarseness.

A change in the local textural situation of an entire image cannot be shown using a GLCM, if spots or minor defects are present on the seed surface. Therefore, the local similarity pattern (LSP) [18] method was used to calculate the textural changes in this study. The LSP method is an algorithm used to determine regional textural changes with rotational invariance. The average gray level and coarseness [19] of the algorithm can be examined to classify seeds. For example, the LSP algorithm can detect defects or uneven parts on the surface of a seed (Figure 8). The surface in Figure 8a has some spots; in Figure 8b, the seed surface is rough, and in Figure 8c, the surface is smooth without obvious spots.

In summary, the textural features of ASM, ENT, CON, HOM, average gray level, and coarseness of the LSP were used to establish a classifier in this study.

### 2.4. Back-Propagation Neural Network Classifer

**Figure 9:**
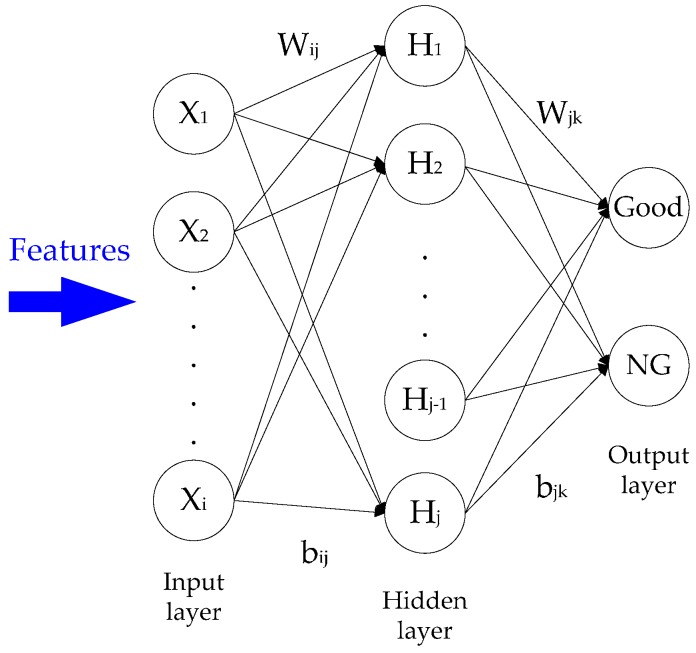
Structure of back-propagation neural network (BPNN) classifier.

In this study, shape, color, and textural features were adopted to classify the quality of Chinese cabbage seeds. A total of 15 shape features (i.e., circularity 1, compactness 1, defect ratio, circularity 2, compactness 2, ellipticity index, elongation, eccentricity, out of roundness, maximum angle of the convex hull, minimum angle of the convex hull, maximum convex distance, symmetry area ratio 1, symmetry area ratio 2, and symmetry area ratio 3), six color features (i.e., mean gray levels on the RGB and HSI bands: R_mean_, G_mean_, B_mean_, H_mean_, S_mean_, and I_mean_), and six texture features (i.e., ASM, ENT, CON, HOM, average gray level, and coarseness of LSP) were employed to classify seed quality. Two back-propagation neural networks (BPNNs) [20] were used to classify seeds as either good or NG. Each BPNN classifier consisted of three layers: an input layer, a hidden layer, and an output layer. The input features were normalized between 0 and 1. The output layer was composed of nodes related to two categories: good and NG. The number of nodes in the hidden layer ($n_{h}$) was calculated using the following formula:

$$n_{h}=0.5(n_{i}+n_{o})$$

where *n_i_* and *n_o_* are the number of input and output nodes, respectively. The structure of the BPNN classifier is illustrated in Figure 9, wherein W_ij_ and b_ij_ are the weight and bias of the input layer in the hidden layer and W_jk_ and b_jk_ are the weight and bias of the hidden layer in the output layer. X_i_, H_j_, and O_k_ denote the input layer, hidden layer, and output layer values, respectively. After its structure was determined, two BPNN classifiers were trained. The purpose of BPNN training is to classify relationships between patterns composed of features in each seed. During training, the BPNN classifier analyzed training samples at a given learning rate, and its weights and biases were adjusted until the mean squared error was less than the tolerance error, which indicated that the BPNN classifier had completed training and its weights and biases were stable. In this study, the BPNN classifier analyzed 2413 training samples at a learning rate of 0.01 before training was complete, as defined by a tolerance error of 0.01.

Dependency or repetition may exist between the various features; therefore, sequential floating forward selection (SFFS) [21] was used to remove some dependent features and reduce the computation of the BPNNs. The quality of the seeds was determined when the output results of two BPNNs were simultaneously good.

## 3. Results

### 3.1. Feature Selection

In this study, the following seed shape features were used to establish a shape classifier with a BPNN: circularity 1 (No. 1), compactness 1 (No. 2), defect ratio (No. 3), circularity 2 (No. 4), compactness 2 (No. 5), ellipticity index (No. 6), elongation (No. 7), eccentricity (No. 8), out of roundness (No. 9), maximum angle of the convex hull (No. 10), minimum angle of the convex hull (No. 11), maximum convex distance (No. 12), symmetry area ratio 1 (No. 13), symmetry area ratio 2 (No. 14), and symmetry area ratio 3 (No. 15). In addition, the following seed color and textural features were adopted to establish a color–texture classifier with the BPNN: $\bar{B}$ (No. 1), $\bar{G}$ (No. 2), $\bar{R}$ (No. 3), $\bar{H}$ (No. 4), $\bar{S}$ (No. 5), $\bar{Gray}$ (No. 6), LSP gray average (No. 7), entropy (No. 8), angular 2nd moment (No. 9), contrast (No. 10), homogeneity (No. 11), and LSP coarseness (No. 12). First, 2801 samples (1197 training and 1704 validation samples) were used to establish a shape BPNN through SFFS. The feature selection results are presented in Table 3. Out of roundness (No. 9) was treated as the basic feature because its accuracy was the highest independently. Ten shape features (Nos. 3, 4, 6, 7, 8, 9, 11, 12, 14 and 15) were selected as the input features of the shape BPNN. The hidden and output layers contained 6 and 2 nodes, respectively. Second, 2413 samples (846 training and 1567 validation samples) were used to establish a color–texture BPNN through SFFS with the basic feature $\bar{Gray}$. The features selection results are presented in Table 4. Nine color and texture features (Nos. 1, 4, 5, 6, 7, 8, 10, 11 and 12) were selected as the input features of the color–texture BPNN. The hidden and output layers contained six and two nodes, respectively. As mentioned, a classifier employing the shape and color–texture BPNNs was established for sorting Chinese cabbage seeds in this study.

### 3.2. Results and Discussion

**Figure 10:**
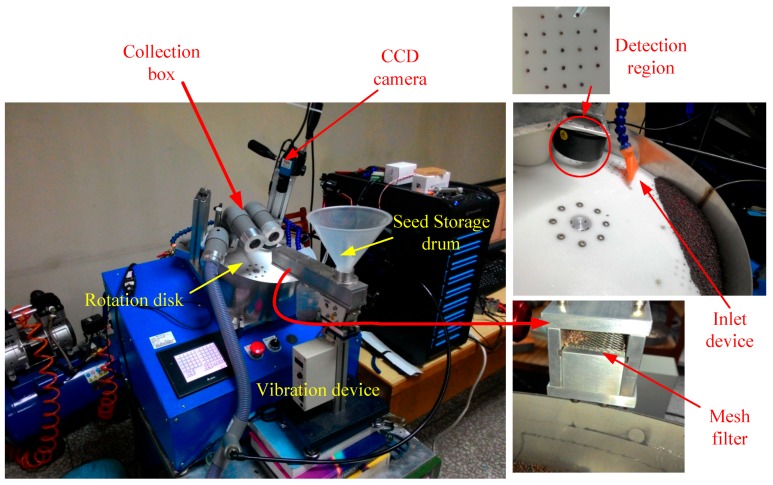
Auto-sorting device for Chinese cabbage seeds.

In this study, an auto-sorting device for Chinese cabbage seeds consisting of a seed storage drum, mesh filter, rotation disk, outlet device, image capture system, collection box, PC and PLC was constructed (Figure 10). The sorting software for Chinese cabbage seeds was developed using Visual Studio 2010-#C and EmguCVx86 2.4.0.1717. The classified seeds were discharged into collection boxes when the solenoid valves were actuated according to the output results of the classifier.

**Figure 11:**
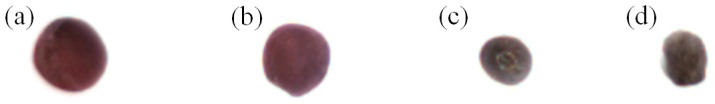
Size: (**a**) excellent; (**b**) excellent; (**c**) small; (**d**) small.

**Figure 12:**
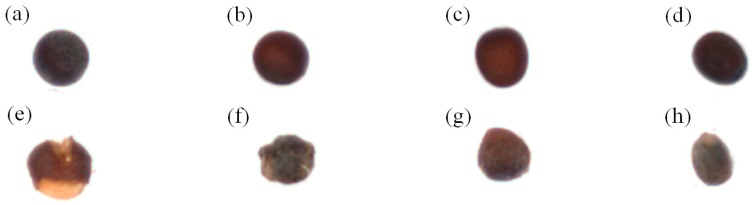
(**a**) Circular; (**b**) circular; (**c**) oval; (**d**) oval; (**e**) irregular; (**f**) irregular; (**g**) triangular; (**h**) elongated.

**Figure 13:**
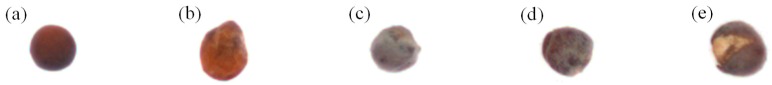
(**a**) Reddish-brown; (**b**) red; (c) white; (**d**) white mist; (**e**) damaged surface.

The constructed system was tested using randomly sampled seeds (8922 good and 7128 NG) separately. The classification accuracies for good and NG seeds were 91.53% and 88.95%, respectively, and the average classification accuracy was 90.38%; the results are shown in Table 5. The speed of sorting was as high as 200 seeds/min, and the time required for extracting all features for the 21 seeds in an image was approximately 601 ms. Sorted good and NG seeds are shown in Figure 11, Figure 12 and Figure 13; however, a few images could not be detected using the proposed algorithms. Examples of classification failure and explanations are provided in Table 6. The system applied in this study could classify the visible side of the seeds by using the CCD camera, but it could not inspect the side hidden from the view of the camera.

The proposed process could classify the quality of Chinese cabbage seeds accurately and efficiently by using the auto-sorting system. This system is a novel implementation device that employs image processing, mechanical devices, software, and a control system. In this study, Chinese cabbage seeds were sorted through a novel approach based on neural networks established using the SFFS algorithm, which analyzed 19 features including shape, color, and texture. In the future, the proposed system can potentially be further developed and self-trained to sort seeds according to the famer’s seed requirements by using BPNN or other classifiers. We hope that this sorting system can be further applied in the future to detect the quality of other seeds.

## 4. Conclusions

In this study, we developed a novel auto-sorting system for Chinese cabbage seeds with an inlet-outlet mechanism, machine vision hardware and software, and a control system for sorting seed quality. The shape, color, and textural features of the seeds were obtained to establish BPNN classifiers. The test results show that Chinese cabbage seeds can be sorted efficiently with this sorting system. In a future study, we intend to further refine the inspection algorithm or use other classifiers (e.g., deep neural networks [22]) to improve the seed inspection accuracy.

## Figures and Tables

**Figure 4:**
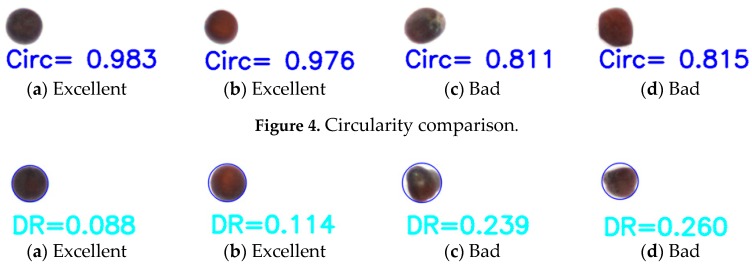
Circularity comparison.

**Figure 5:**
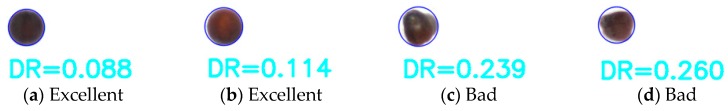
Defect ratio comparison.

**Figure 6:**
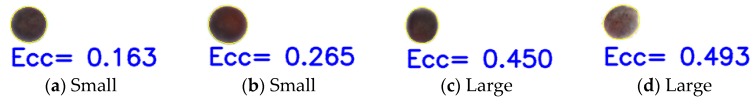
Eccentricity comparison.

**Figure 7:**
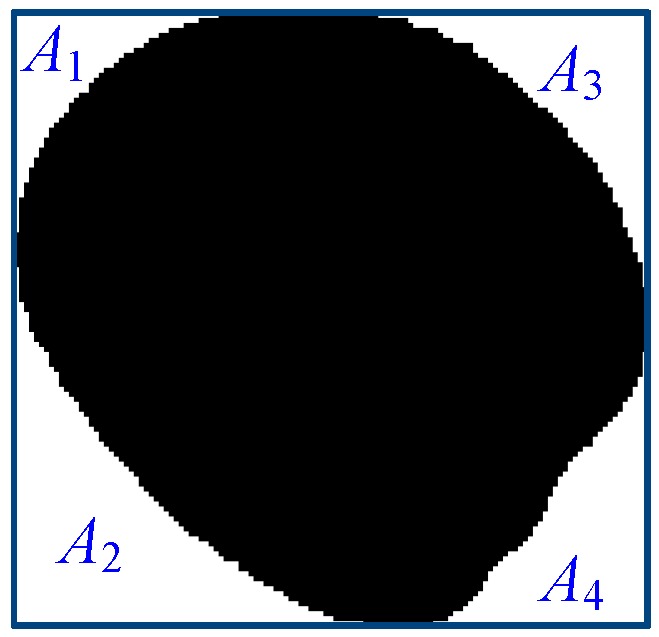
Symmetrical area ratio.

**Table 1:** Mathematical formulations of shape features.

Feature	Formulation	Diagram
Compactness 1	$\frac{2\sqrt{A_{Circle}\pi }}{P}$	~
Compactness 2	$\sqrt{\frac{4A}{\pi }}/D_{\max}$	~
Circularity 1	$4\pi \times A/P^{2}$	~
Circularity 2	$\sqrt{\frac{4A}{\pi {D_{\max}}^{2}}}$	Figure 4
Defects ratio	$\frac{A_{Cricum}-A}{A_{Cricum}}$	Figure 5
Elongation	$D_{\min}/D_{\max}$	~
Ellipticity index	$\pi \times a^{2}/A$	~
Eccentricity	$\sqrt{a^{2}-b^{2}}/a$	Figure 6
Symmetry area ratio 1	$\left|1-\frac{A{\;}_{1}+A{\;}_{2}}{A{\;}_{3}+A{\;}_{4}}\right|$	Figure 7
Symmetry area ratio 2	$\left|1-\frac{A{\;}_{1}+A{\;}_{3}}{A{\;}_{2}+A{\;}_{4}}\right|$	Figure 7
Symmetry area ratio 3	$\left|1-\frac{A{\;}_{1}+A{\;}_{4}}{A{\;}_{2}+A{\;}_{3}}\right|$	Figure 7

Note: *A_circle_* is the circle area that is the same as the seed area, *P* is the seed perimeter, *A* is the seed area, *D_max_* is the largest diameter of the object, *D_min_* is the smallest diameter of the object, *A_Cricum_* is the minimum circumcircle area, *a* is the semimajor axis, *b* is the semiminor axis, and *A*_1_–*A*_4_ are symmetrical rectangular areas (Figure 7).

**Table 2:** Mathematical formulations of GLCM features.

Feature	Formulation
Angular 2nd moment	$\sum_{i=1}^{N_{x}}\sum_{j=1}^{N_{x}}{\left[P(i,j,d,\theta )\right]}^{2}$
Entropy	$\sum_{i=1}^{N_{x}}\sum_{j=1}^{N_{x}}P(i,j,d,\theta )\log P(i,j,d,\theta )$
Contrast	$\sum_{i=1}^{N_{x}}\sum_{j=1}^{N_{x}}{\left(i-j\right)}^{2}P(i,j,d,\theta )$
Homogeneity	$\sum_{i=1}^{N_{x}}\sum_{j=1}^{N_{x}}\frac{1}{1+{\left(i-j\right)}^{2}}P(i,j,d,\theta )$

**Table 3:** Selection of shape features.

Number	Accuracy	Feature Subset
(1)	76.46%	{9}
(2)	85.38%	{9, 8}
(3)	85.79%	{9, 8, 3}
(4)	88.02%	{9, 8, 3, 12}
(5)	89.30%	{9, 8, 3, 12, 1}
(6)	89.26%	{9, 8, 3, 12, 1, 11}
(7)	89.50%	{9, 8, 3, 12, 11, 14}
(8)	91.61%	{9, 8, 3, 12, 11, 14, 1}
(9)	90.49%	{9, 8, 3, 12, 11, 14, 1, 13}
(10)	90.72%	{9, 8, 3, 12, 11, 14, 1, 13, 10}
(11)	90.14%	{9, 8, 3, 12, 11, 14, 1, 13, 10, 4}
(12)	90.90%	{9, 8, 3, 12, 11, 14, 1, 13, 10, 4, 7}
(13)	91.00%	{9, 8, 3, 12, 14, 1, 13, 10, 4, 7, 15}
(14)	91.10%	{9, 8, 3, 12, 14, 1, 13, 4, 7, 15}
(15)	90.90%	{9, 8, 3, 12, 14, 1, 4, 7, 15}
(16)	91.61%	{9, 8, 3, 12, 14, 1, 4, 7, 15, 11}
(17)	91.31%	{9, 8, 3, 12, 14, 1, 4, 7, 15, 11, 2}
(18)	91.70%	{9, 8, 3, 12, 14, 4, 7, 15, 11, 2, 6}
**(19)**	**92.00%**	**{9, 8, 3, 12, 14, 4, 7, 15, 11, 6}**
(20)	91.00%	{9, 8, 3, 12, 14, 7, 15, 11, 6}
(21)	91.00%	{9, 8, 3, 12, 14, 7, 15, 11}
(22)	91.66%	{9, 8, 3, 12, 14, 7, 15, 11, 5}
(23)	91.70%	{9, 8, 3, 12, 14, 15, 11, 5, 2}
(24)	91.80%	{9, 8, 3, 12, 14, 15, 11, 2, 10}
(25)	91.25%	{9, 8, 3, 12, 14, 15, 11, 2, 10, 4}
(26)	91.90%	{9, 8, 3, 12, 14, 15, 11, 2, 10, 4, 1}
(27)	91.55%	{9, 8, 3, 12, 14, 15, 11, 2, 10, 4, 1, 6}

**Table 4:** Selection of color and textural features.

Number	Accuracy	Feature Subset
(1)	68.28%	{6}
(2)	71.34%	{6, 12}
(3)	73.83%	{6, 12, 7}
(4)	75.62%	{6, 12, 7, 5}
(5)	77.70%	{12, 7, 5, 4}
(6)	79.38%	{12, 7, 5, 4, 10}
(7)	81.68%	{12, 7, 5, 4, 10, 6}
(8)	82.51%	{12, 7, 5, 4, 10, 6, 8}
(9)	84.20%	{12, 7, 5, 4, 10, 8, 11}
(10)	84.30%	{12, 5, 4, 10, 8, 11}
(11)	81.70%	{5, 4, 10, 8, 11}
(12)	79.20%	{5, 4, 8, 11}
(13)	76.50%	{4, 8, 11}
(14)	78.49%	{4, 8, 11, 5}
(15)	79.50%	{4, 8, 5, 9}
(16)	82.06%	{4, 8, 5, 9, 12}
(17)	83.47%	{4, 8, 5, 9, 12, 11}
(18)	82.10%	{4, 8, 5, 9, 12, 11, 7}
(19)	82.40%	{4, 8, 5, 9, 12, 11, 6}
(20)	84.20%	{4, 8, 5, 12, 11, 6, 10}
(21)	85.25%	{4, 8, 5, 12, 11, 6, 10, 2}
(22)	85.32%	{4, 8, 5, 12, 11, 6, 10, 2, 7}
(23)	85.40%	{4, 8, 5, 12, 11, 10, 2, 7, 1}
**(24)**	**87.20%**	**{4, 8, 5, 12, 11, 10, 7, 1, 6}**
(25)	85.96%	{4, 8, 5, 12, 11, 10, 7, 1, 6, 3}

**Table 5:** Results.

Type	Good	NG
Good	8166	788
NG	756	6340
Accuracy	91.53%	88.95%

**Table 6:** Examples of classification failure and explanations.

	Image	Explanation
Case 1	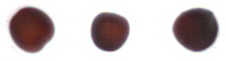	The shapes are similar to circular, but the captured images show hollow, square or triangle because of the seed placement angle.
Case 2	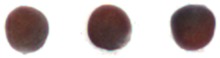	The shapes are similar to triangular or irregular, but the captures images are similar to circular.
Case 3	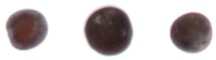	The other sides of the seeds are damaged or show defects.
